# Are there associations between clinical and embryological factors with pregnancy loss following transfer of a single euploid embryo?

**DOI:** 10.1530/RAF-24-0002

**Published:** 2024-07-02

**Authors:** Beril Yuksel, Gonul Ozer, Ipek Nur Balin Duzguner, Aysu Akca, Yesim Kumtepe, Hakan Yelke, Semra Kahraman, George Liperis, Munevver Serdarogullari

**Affiliations:** 1 Istanbul Sisli Memorial Hospital, Department of ART and Reproductive Genetics, Istanbul, Türkiye; 2 Westmead Fertility Centre, Institute of Reproductive Medicine, University of Sydney, Westmead, NSW, Australia.; 3 Department of Histology and Embryology, Faculty of Medicine, Cyprus International University, Northern Cyprus via Mersin 10, Türkiye

**Keywords:** euploid embryo transfer, recurrent pregnancy loss, morphokinetic parameters, time-lapse imaging, preimplantation genetic testing, frozen embryo transfer

## Abstract

First-trimester pregnancy losses are commonly attributed to chromosomal abnormalities. The causes of pregnancy loss following transfer of a euploid embryo are not fully elucidated. The aim of this study was to evaluate clinical and embryological parameters for pregnancy failure following the transfer of a single euploid embryo. Pregnancy outcomes of single euploid embryo transfers from a single centre between January 2017 and March 2020 were retrospectively evaluated. Several clinical and embryological parameters were evaluated in consideration to pregnancy outcomes; total pregnancy loss and live birth (LB). Endometrial preparation type, number of previous frozen embryo transfer cycles, history of recurrent pregnancy loss, higher body mass index, presence of endometriosis and/or adenomyosis and embryo quality were found to be significantly different between two groups. Morphokinetic parameter analysis of 523 euploid embryos using time-lapse imaging did not show any statistical differences between the two groups; however, a significantly higher rate of uneven blastomeres in the cleavage stage was observed in the total pregnancy loss group. Evaluation of clinical and embryological data can reveal possible factors associated with pregnancy loss that can facilitate improved patient consultation. Feasible interventions can potentially increase the chance of achieving an LB.

**Lay Abstract:**

Like natural pregnancies, not all pregnancies following fertility treatment go to term. The most common reason for these losses is that these embryos lack the genetic constitution compatible with live birth. Combined with fertility treatment, genetic tests can evaluate the genetic ability of embryos to go to term. Monitoring the outcome of pregnancies resulting from such embryos can help us identify whether and which conditions specific to treatment can lead to pregnancy loss. The analysis identified four parameters associated with embryo loss: Embryo quality and division patterns, existence of previous treatment and treatment type.

## Introduction

Preimplantation genetic testing for aneuploidy (PGT-A) is widely used in assisted reproductive technologies (ART) to detect chromosomal abnormalities in embryos and it is utilised for various indications such as advanced maternal age (AMA), severe male factor (SMF), recurrent implantation failure (RIF) and recurrent pregnancy loss (RPL). This method is used to maximise chances of live birth rate (LBR), while minimising the risk of clinical miscarriage, futile transfers and ongoing aneuploid pregnancies, ultimately leading to reduced time to reach a live birth (LB). Nonetheless, the LBR after a euploid embryo transfer are still far from the desired success rates.

A spontaneous miscarriage is estimated to occur in 12−15% of all pregnancies (de Ziegler & Frydman 2021). Although there are many clinical factors reported to have an association with a spontaneous miscarriage; chromosomal abnormalities of the embryo represents the major cause of first-trimester pregnancy losses (Levy *et al*. 2014, Bulletins—Gynecology 2015). In the era of ART and in spite of the advantage of PGT-A in preventing miscarriages, a pregnancy loss still can occur after the transfer of a single chromosomally normal embryo. Efforts to improve LBR in ART have led the researchers to investigate the factors that cause a pregnancy loss, other than chromosomal abnormalities. In the last few decades, there have been many studies reporting the effects of endometriosis, adenomyosis, chronic endometritis, obesity, endocrine and autoimmune disorders, and structural anatomical problems on pregnancy losses (Luke 2017, Alecsandru *et al*. 2021, de Ziegler & Frydman 2021, Pirtea *et al*. 2021). The association between pregnancy outcome and preimplantation embryonic development have also been investigating with the use of time-lapse systems (TLSs), which enables continuous monitoring of developmental stages and morphokinetic parameters of embryos (Motato *et al*. 2016, Rienzi *et al*. 2019, McQueen *et al*. 2021). Studies using TLS have have reported conflicting outcomes with some showing altered morphokinetic division times in embryos resulting in pregnancy loss, whereas others not identifying any significant differences. Recent advances in artificial intelligence (AI) analysing TLS-generated images have also been introduced that can be used to select embryos that have higher chances to implant and result in LB (Tran *et al*. 2019, Friedenthal *et al*. 2021, Sawada *et al*. 2021).

The main question clinicians face when counselling a patient after a euploid pregnancy loss is related to identification of a probable cause that lead to the miscarriage and advice on what can be done next to avoid similar outcome. Therefore, the aim of this study was to examine if there are any clinical and/or embryological factors that can be associated with pregnancy loss following transfer of a single euploid embryo.

## Materials and methods

### Patients and setting

All positive pregnancies following a single euploid embryo transfer between January 2017 and March 2020 were retrospectively evaluated. This study was undertaken at a single centre (Istanbul Memorial Hospital) and the institutional review board of Istanbul Memorial Hospital, 24.12.2021/008, approved the study. All pregnancies were followed and reported outcomes in terms of pregnancy loss and LB. The cut-off for female age for undergoing treatment with autologous oocytes was 43 years of age. Indications for PGT-A included AMA, history of RPL, RIF, history of abnormal fetal karyotype, and patient choice for reducing time to reach pregnancy. The diagnosis of RPL was made following the occurrence of two or more pregnancy losses, which may result from spontaneous conception or ART, while excluding ectopic and molar pregnancies. The definition of RIF was as follows: failure to achieve implantation in a couple after two transfers with good-quality blastocysts.

All patients undergoing oocyte pick-up (OPU) were evaluated for uterine, endometrial and tubal anatomic abnormalities and endocrine disorders including thyroid dysfunction, hyperprolactinaemia and diabetes, while semen analysis was conducted for all male partners. Exclusion criteria consisted of patients with structural rearrangements such as inversions and translocations, patients with endometrial factor which was defined as inadequate endometrial thickness in the frozen embryo transfer (FET) cycle (<7 mm), cases with anatomic malformations, untreated endocrine disorders, azoospermia, and sperm not produced by ejaculation.

Included cases were divided into two groups: total pregnancy loss (TPL) and LB group according to pregnancy outcomes. TPL was defined as pregnancies that did not result in a LB constituting of biochemical and clinical pregnancy losses.

The analysis included clinical features such as maternal age, body mass index (BMI) value, anti-Müllerian hormone (AMH) levels, duration of infertility, number of previous ART and FET cycles, presence of adenomyosis and/or endometriosis, presence of polycystic ovarian syndrome (PCOS), severe male factor defined by total motile sperm count of less than 5 million, and history of RPL or RIF. The indications for PGT-A and cycle characteristics such as total number of oocytes retrieved, total number of mature and fertilised oocytes, type of endometrial preparation for FET cycles, and maximum endometrial thickness in FET cycles were also included in the analysis. In terms of embryological data, the quality of the embryos transferred was compared between the two groups. Since the use of TLI was not available for all patients, assessment of morphokinetic parameters of euploid embryos using TLI were compared in a sub-group analysis consisting of 523 embryos between groups of TPL and LB.

### Controlled ovarian hyperstimulation

A controlled ovarian stimulation with short antagonist protocol was used for all cycles. Stimulation initiated on the second day of menstruation with initial dosages adjusted for age, BMI, antral follicle count, and history obtained from previous ART cycles. The choice of the gonadotrophins were either recombinant FSH (Gonal-F 450 IU/0.75 mL, Merck Serono, Switzerland), recombinant LH (Luveris, 75 IU, Merck Serono, Switzerland) recombinant FSH+LH (Pergoveris 150 IU/75 IU, Merck Serono, Switzerland) or human menopausal gonadotrophins (Menogon, Ferring, Istanbul, Türkiye, and Menopur, Ferring, Istanbul, Türkiye). Dosages were adjusted between 75 and 600 IU/day according to serial monitorisation of follicular development and hormonal assays. An antagonist (Cetrotide, 250 µg, Merck Serono) was administered daily when the measurement of leading follicle was 12–13 mm. Depending on the risk of ovarian hyperstimulation, a single dosage of 250 µg recombinant hCG (Ovitrelle amp 250 µg/0.5 mL, Merck Serono) or 0.2 mg of triptoreline acetate (Gonapeptyl 0.1 mg/mL, Ferring) was administered as a trigger injection when at least three follicles reached a mean diameter of >18 mm. Transvaginal US-guided oocyte retrieval was performed approximately 36 h after trigger injection. Fertilisation of the oocytes was performed using standard intracytoplasmic sperm injection (ICSI) techniques.

### Time-lapse imaging

Following ICSI, injected oocytes were either positioned in a time-lapse incubator (EmbryoScope™) or a benchtop incubator at 6% CO_2_, 5% O_2_, and 37°C for 5 days. When Embryoscope was used, images were acquired every 15 min, and data were continuously transferred to an external computer, EmbryoViewer® workstation (Vitrolife, Göteborg, Sweden).

The morphokinetic parameters were annotated by a single senior embryologist (YK) with several years of experience to avoid inter-observed variability. The time to reach each morphokinetic stage, the presence of direct or reverse cleavage, and the presence of uneven blastomere sizes were recorded for the two groups. Blastomere size is dependent on both the cleavage stage and the regularity of each cleavage division and blastomeres of two cells, four cells, and eight cells from embryos at each stage are expected to have equal cell size among them. Evaluated morphokinetic stages consisted of time to pronucleate (PN) appearance (tPNa), time to PN fading (tPNf), time to two cells (t2), time to three cells (t3), time to four cells (t4), time to eight cells (t8), time to nine cells (t9), time to start of compaction (tSC), time to morula (tM, defined as the full compaction state), time to start of blastulation (tSB, defined as the initiation of a cavity formation), time to blastocyst (tB), and time to expanded blastocyst (tEB, defined as 50% thinning of the zona pellucida). The time points were measured in hours from the time of ICSI.

### Embryo morphological grading

Blastocysts were scored according to Gardner’s blastocyst scoring classification, with the evaluation of embryo expansion, and scoring of inner cell mass (ICM) and trophoectoderm (TE). Blastocysts were classified into three groups: top quality (TQ), good quality (GQ), and poor quality (PQ). The TQ embryos included 3-6 AA blastocysts, whereas GQ embryos included 3-6 BB, AB, or BA blastocysts as per the Gardner grading system. Blastocysts of inferior quality were defined as PQ blastocysts (Kahraman *et al*. 2020).

### Trophoectoderm biopsy

Zona pellucida opening was performed on the third day of embryo development, using a diode laser (RI Saturn 3, England). On the fifth day of development, five to eight TE cells were obtained from the developed blastocyst by either the use of laser or by mechanical detachment using the biopsy and the holding pipette. Following visualisation of re-expansion, blastocysts were vitrified using Kitazato vitrification media (Kitazato, Japan) as per manufacturer’s instructions.

### Next-generation sequencing (NGS)

The DNA was extracted from biopsied material and whole genome amplification (WGA) was performed using an Ion Torrent Ion SingleSeq™ 96 kit (Thermo Fisher Scientific). The next-generation sequencing procedure was completed using the Ion Chef System (Thermo Fisher Scientific) and Ion GeneStudio S5 (Thermo Fisher Scientific). Data analyses were performed with Ion Reporter Software v5.6 (Thermo Fisher Scientific).

### Frozen embryo transfer

Two types of endometrial preparation were used based on cycle characteristics of the patients. Modified natural (MN) endometrial preparation was preferred for patients with ovulatory menstrual cycles, whilst endometrial preparation with sequential estrogen and progesterone (hormone replacement therapy – HRT) was used for patients with non-ovulatory menstrual cycles.

In particular, the type of endometrial preparation was determined based on the patients’ menstrual cycles. Patients with regular menstrual cycles underwent a natural cycle for endometrial preparation. However, if a patient experienced two consecutive menstrual cycles without ovulation, a hormonal replacement cycle was initiated. For ovulatory patients, the natural cycle was always the preferred choice for endometrial preparation unless the patient objected. Conversely, for patients without regular ovulatory cycles, endometrial preparation was achieved using sequential estrogen and progesterone. The estrogen dose was fixed at 6 mg/day for a minimum of 14 days, which could be extended up to 21 days if the endometrial thickness was deemed inadequate. On the 15th day, progesterone vaginal gel (Crinone 8%; Merck Serono) was initiated twice daily, alongside the existing estrogen dose.

For both endometrial preparation methods, ultrasonographic evaluation was carried out on the second day of menstruation to rule out the presence of any ovarian cysts or other pelvic pathologies. In the MN cycle, the ultrasonographic examination was repeated on day 9 or 10, depending on the cycle length, to observe whether there was a spontaneously growing dominant follicle. When a dominant follicle with a size of at least 15 mm was observed, serum levels of estradiol and LH were monitored on a daily basis. A trigger injection with recombinant hCG (Ovitrelle, Merck Serono) was administered at any time between the onset of LH surge (15 IU/L) and LH peak (≥40 IU/L), as previously described (Kahraman & Sahin 2020).

In HRT, estrogen patches (3.9 mg estradiol transdermal patch, Climara patch, Bayer Turk, Türkiye) or oral estradiol tablets (2 mg three times per day – total 6 mg) (Estrofem, Novo Nordisk) were administered on the second day of menstruation. Ultrasonographic examination was repeated on day 11 and day 15 of the cycle to monitor endometrial thickness and to observe whether there was a spontaneously growing dominant follicle. If there was a dominant follicle, the cycle continued as a natural cycle. When an adequate endometrial thickness of ≥7 mm and above was observed on day 15, progesterone vaginal gel (Crinone 8%; Merck Serono) was administered twice a day. If endometrial thickness was not observed on day 15, estrogen administration continued to day 21.

The embryo transfer was carried out 5 days after the commencement of progesterone administration. On the day of transfer, embryo warming was performed using Kitazato warming media (Kitazato, Japan) as per manufacturer’s instructions. Embryos were checked for re-expansion, vitality, and necrotic foci at 30 min, 2 h and 4 h after warming at which time the embryo transfer took place. Luteal support with vaginal progesterone was continued until the 10th week of pregnancy.

A serum βhCG level above 20 IU/L after 9 days of embryo transfer was considered a positive pregnancy. A clinical pregnancy was defined as the presence of a fetal sac visualised by ultrasound examination at 6–8th weeks. Pregnancies that continued for >12 weeks were considered as ongoing pregnancy and LB was defined as the live born after 24 weeks of pregnancy.

Biochemical pregnancy loss (BPL) was defined as the declining of βhCG level after a positive pregnancy. Clinical pregnancy loss (CPL) was defined as pregnancy loss after the visualisation of an intrauterine gestational sac by ultrasound. TPL was defined as pregnancies that did not result in LB, consisting of both biochemical and clinical pregnancy losses.

### Statistical analysis

Statistical analyses were performed using the SPSS (version 22). Categorical variables were presented as counts and percentages, and continuous variables were presented as mean ± s.d. or median and interquartile range, as appropriate. Shapiro–Wilk test was performed to test the distribution of the variables. ANOVA (analysis of variance), Kruskal–Wallis, *χ* ^2^-test, and Fisher–Freeman–Halton tests were used for comparison of the groups. The covariates with *P* < 0.05 were included in backward-step logistic regression analysis in order to define independent factors associated with pregnancy loss after a euploid embryo transfer. A *P*-value ≤ 0.05 was considered significant.

## Results

### Clinical outcomes

The data consisted of 1492 pregnancies obtained as a result of the transfer of 2041 single euploid embryos within the study time-frame. The total pregnancy rate was 73.1% (1492/2041). From the 1492 pregnancies that were included in the study, 299 resulted in pregnancy loss (20.0%, 299/1492), whilst 1193 resulted in LB (58.4%, 1193/2041). From the 299 patients experiencing TPL, 145 were due to BPL (9.7%, 145/1492) and 154 were due to CPL (11.4%, 154/1347). Indications for PGT-A were as follows: AMA (28.6%), RPL (14.9%), and RIF (11.7%), the monogenic disease carriers who requested PGT-A in addition to PGT-M and who has history of abnormal fetal karyotype (12.1%) and other reasons (32.6%).

### Clinical/cycle characteristics and pregnancy outcomes

The clinical and cycle characteristics of 1492 patients that resulted in positive pregnancy were analysed for the two groups with 299 pregnancies resulting in TPL and 1193 resulting in LB (Table 1). No statistical differences in terms of female age, serum AMH level, duration of infertility, mean numbers of oocytes retrieved, number of mature oocytes and number of fertilised oocytes (*P* > 0.05, Table 1). BMI values were significantly lower in LB group when compared to TPL (*P* < 0.001, Table 1). Similarly, the number of previous OPU and FET cycles and the presence of severe endometriosis and/or adenomyosis were significantly lower in the LB group (*P* < 0.001 and *P* < 0.021, respectively, Table 1). The history of RPL was more frequent in the TPL group (*P* = 0.004, Table 1). On the other hand, the presence of polycystic ovarian syndrome (PCOS), severe male factor or history of RIF did not differ among groups (*P* > 0.05, Table 1). When TPL was evaluated according to PGT-A indications, history of RPL had the highest rate (27.8%) compared to AMA (19.6%), RIF (19.4%), abnormal karyotype (21.6%), and patient choice (16.4%, *P* < 0.05).

**Table 1:** Clinical and cycle characteristics of the patients according to pregnancy outcome. Data are presented as median (minimum–maximum) and percentages.

	Total pregnancy loss	Live birth	*P*
*n*	299	1193	
AMH (ng/mL)	2.40 (0.01–15.40)	2.35 (0.03–19.59)	0.573
Age	35.00 (21–42)	35.00 (20–42)	0.548
BMI (kg/m^2^)	24.10 (17.0–42.0)	23.50 (15.0–42.2)	0.001*
Duration of infertility (years)	4 (1–22)	4 (1–25)	0.550
Number of previous OPU cycles	5 (2–18)	4 (2–17)	<0.001*
Number of previous FET cycles	2 (1–7)	1 (1–8)	<0.001*
Number of oocytes retrieved	12 (1–46)	11 (1–46)	0.145
Number of mature oocytes	10 (1–34)	10 (1–44)	0.391
Number of fertilised oocytes	9 (0–32)	8 (1–39)	0.459
History of recurrent pregnancy loss	26.8%	19.3%	0.004*
History of recurrent implantation failure	21.4%	18.9%	0.336
Presence of endometriosis/adenomyosis	4.7%	2.1%	0.021*
PCOS	9.0%	8.7	0.864
Severe male factor	25.1%	24.4%	0.804
Type of endometrial preparation			<0.001*
Natural	47.0%	74.6%	
Hormone replacement	53.0%	25.6%	
Embryo quality			0.019*
TQ	56.2%	64.8%	
GQ	42.1%	34.2%	
PQ	1.7%	1.0%	

*P < 0.05 is considered statistically significant.

AMH, anti-Müllerian hormone;BMI, body mass index; FET, frozen embryo transfer; GQ, good quality; PCOS, polycystic ovarian syndrome; PQ, poor quality; TQ, top quality.

### Type of FET cycle and pregnancy outcomes

The pregnancy outcome comparison for MN and HRT cycles is shown in Table 2. In the TPL group, the percentage of HRT cycles was higher (*P* < 0.001). The overall evaluation of pregnancy results in HRT and MN cycles also revealed a significant increase in miscarriage rates in HRT cycles (*P* < 0.001, Table 2).

**Table 2:** Pregnancy outcomes in natural and hormone replacement cycles. Data are presented as percentages.

	MN cycle (*n* = 1387)	HRT cycle (*n* = 654)	*P*
βhCG+	74.69%	71.1%	0.09
BPL	7.82%	13.8%	<0.001*
CPL	6.28%	23.7%	<0.001*
Live birth	64.53%	46.8%	<0.001*

*P < 0.05 is considered statistically significant.

BPL, biochemical pregnancy loss; CPL, clinical pregnancy loss; HRT, hormone replacement; MN, modified natural.

### Embryo quality and pregnancy outcomes

When embryo quality was compared for the two groups, TQ embryos constituted the majority of embryos in both LB and TPL groups (64.8 and 56.2 respectively), followed by GP (34.2 and 42.1 respectively) and PQ (1.0 and 1.7 respectively, Table 3). Top embryo quality demonstrated a significant association with LB with good-quality embryos more likely to result in TPL (*P* = 0.019, Table 3).

**Table 3:** Embryo quality and pregnancy outcome.

Embryo quality	Total pregnancy loss (*n* = 299)	Live birth (*n* = 1193)	*P*
			0.019*
Top quality	56.2%	64.8%	
Good quality	42.1%	34.2%	
Poor quality	1.7%	1.0%	

### Morphokinetic/embryological parameters and pregnancy outcomes

The morphokinetic parameters of 523 euploid embryos were analysed according to pregnancy outcomes (Table 4). The time to reach each morphokinetic stage and the presence of direct or reverse cleavage were not different between the two groups (*P* > 0.05). A significantly higher rate of uneven blastomeres in cleavage stage embryos was observed in the TPL group (*P* = 0.036, Table 4).

**Table 4:** Morphokinetic parameter comparison for different pregnancy outcomes. Data presented as mean ± s.d., or median (minimum–maximum) hours after ICSI depending on the normality distribution of the values with *P* < 0.05 considered as statistically significant.

	Total pregnancy loss	Live birth	*P*
tPNa	8.08 (5.15–13.90)	8.02 (4.25–14.71)	0.763
tPNf	23.46 (19.26–34.41)	23.08 (17.66–32.32)	0.582
t2	25.92 (21.76–37.16)	25.46 (20.43–45.47)	0.260
t3	37.04 (25.76–51.24)	36.87 (24.09–52.67)	0.582
t4	38.31 (29.26–51.24)	37.91 (25.14–66.93)	0.345
t5	50.04 (34.09–69.64)	49.56 (28.45–67.94)	0.903
t6	51.67 (36.66–70.39)	51.50 (34.06–72.70)	0.243
t7	54.34 (36.66–70.39)	53.45 (37.10–78.81)	0.382
t8	56.17 (45.76–74,34)	55.12 (44.64–80.67)	0.313
t9	66.18 (48,34–91,58)	68.16 (46.21–93.67)	0.309
tSC	80.58 (56.97–102.48)	79.62 (54.42–108.66)	0.278
tM	89.17 (71.23–110.98)	88.15 (67.71–119.67)	0.281
tSB	97.99 (84.54–120.41)	97.03 (77.99–126.09)	0.120
tB	104.70 (88.58–123.71)	104.02 (84.75–130.09)	0.178
tEB	110,85 (98.29-130.67)	110.43 (94.09–140.34)	0.380
Presence of uneven blastomere	16.5% (16/97)	8.7% (37/424)	0.036*
1–>3 direct cleavage	10.3% (10/97)	5.9% (25/426)	0.176
2–>5 direct cleavage	9.1% (1/11)	10.2% (42/411)	1.000
Reverse cleavage	1.0% (1/97)	2.3% (10/426)	0.698

tPNa, time to Pn appearance; tPNf, time to Pn fading; t2, time to two cells; t3 time to three cells; t4, time to four cells; t8, time to eight cells; t9, time to nine cells; tSC, time to start of compaction; tM; time to morula; tSB, time to start of blastulation; tB, time to blastocyst; tEB, time to expanded blastocyst, 1–>3 direct cleavage: direct cleavage from 1 to 3 cells, 2–>5 direct cleavage, direct cleavage from 2 to 5 cells.

### Significance of factors in predicting pregnancy loss

After evaluating all factors in the univariate analysis, the factors that were found to be statistically different for TPL were further evaluated using backward-step logistic regression analysis (Table 5). The type of endometrial preparation, the presence of uneven blastomeres, embryo grade, and the number of previous FET cycles were identified to be more likely associated in pregnancy loss after transfer of a euploid embryo (odds ratios of 2.82, 2.09, 1.89, and 1,59 respectively, Table 5). As a result of the *post* *hoc* power analysis using the G Power 3.1 statistical software program, the power for an effect size of 0.2 and a significance level of 0.05 was calculated as 87%.

**Table 5:** Backward-step logistic regression analysis to determine importance of factors contributing to total pregnancy loss after transfer of a single euploid embryo. *P* < 0.05 is considered as statistically significant.

	*P*	OR	95% CI	
			Lower	Upper
Endometrial preparation*: HRT vs NC	<0.001	2.82	1.68	4.75
Morphokinetic evaluation**: UE vs EC	0.045	2.09	1.02	4.31
Embryo grade***: TQ vs GQ	0.011	1.89	1.15	3.08
Number of previous FET cycles	<0.001	1.59	1.30	1.94

*Natural cycle; **Even cleavage; *** TQ embryo.

EC, even cleavage; FET, frozen embryo transfer; GQ, good quality; HRT, hormone replacement; NC, natural cycle; OR, odds ratio; TQ, top quality; UE, uneven.

## Discussion

The most common cause for first-trimester pregnancy losses is chromosomal aneuploidy (Stephenson *et al*. 2002, Levy *et al*. 2014, Bulletins—Gynecology 2015). However, the factors that cause a pregnancy loss after a chromosomally normal embryo transfer are not fully understood. The results of this study have shown that there are multiple factors ranging from patient and cycle characteristics, type of FET cycle, and embryological parameters that may have an impact on pregnancy outcomes following embryo transfer of a single euploid embryo.

In terms of clinical and cycle characteristics, patients with a history of RPL exhibited the highest rate of TPL compared to other factors. It is generally considered that a notable portion of RPL cases is attributed to chromosomal abnormalities. For those patients, PGT-A is known to reduce pregnancy (Bhatt *et al*. 2021). However, identifiable causes for RPL extend beyond ploidy for a number of patients. In this study, we have shown that even following a euploid embryo transfer, after controlling and treating well-known causes of RPL, such as endocrine disorders and anatomical abnormalities, miscarriage rate is still higher than expected in these cases. Maternal age is known to affect success rate following euploid embryo transfer as shown by a recent meta-analysis (Vitagliano *et al*. 2023). Nonetheless, in our study there was a cut-off age of 43 for inclusion and there was no difference in maternal age for the groups of LB and TPL.

The type of endometrial preparation was also found to be associated with pregnancy outcomes as we observed a significant increase in miscarriage rates in HRT cycles compared to MN cycles. This demonstrates that in this particular clinical setting, the endometrial preparation appears to play an important role in the outcome of euploid embryo transfer. The type of endometrial preparation for particular patient groups and HRT protocols may differ in different clinical settings and therefore the results observed might not be corroborated in alternate clinical settings. There are many reports in the literature that support improved pregnancy rates in natural cycles, in which the endometrium is prepared with endogenous estradiol and progesterone secreted from the dominant follicle (Morozov *et al*. 2007, Li *et al*. 2021, Wu *et al*. 2021). Suboptimal hormone levels in artificial cycles have been associated with higher miscarriage rates (Li *et al*. 2021) and it has been suggested that the excessive hormonal stimulation or abnormal estrogen/progesterone ratio in artificial cycles may cause a shift in normal window of implantation (Morozov *et al*. 2007). It is also possible that thromboembolic events induced by excessive hormones may impact placentation and miscarriage (Wu *et al*. 2021). Another hypothesis is the fact that non-ovulatory patients are often older patients or patients with PCOS, something that may have an impact on the results (Wu *et al*. 2021). In our study, both the presence of PCOS and female age were similar between LB and TPL groups.

In terms of embryological observations, embryo quality was shown to affect pregnancy outcome in accordance with the literature (Wang *et al*. 2019, McQueen *et al*. 2021). In the LB group, a higher rate of transfers with TQ embryos was observed. In addition, when overall pregnancy outcomes were evaluated, positive pregnancy rates were found to be higher when the transfer was performed with TQ embryos.

The morphokinetic parameters of 523 euploid embryos, in which the TLI was used, revealed that the time to reach each morphokinetic stage was not significantly different between LB and TPL. In the literature, there is conflicting evidence with some studies reporting altered TLI parameters associated with blastocyst formation, implantation and live birth (Motato *et al*. 2016, Rienzi *et al*. 2019), and a recent study showing no differences (McQueen *et al*. 2021). In terms of other embryological observations from TLI, the presence of direct or reverse cleavage were not different between the LB and TPL groups. However, when compared to the TPL group, a significantly lower rate of uneven blastomeres was observed in the cases that reached to LB. The finding of uneven blastomere size during embryo development and its association with aneuploidies has been shown in other studies (Hardarson *et al*. 2001, Magli *et al*. 2007). The finding of this study shows that uneven blastomeres during the cleavage stage can also result from euploid embryos and this may serve as a risk factor for pregnancy loss. In literature, it has been hypothesised that uneven blastomeres may be an indication of improper mitosis and may cause uneven distribution of cellular material, namely proteins, and organelles and therefore may affect developmental potential of the embryo and therefore pregnancy outcomes (Rienzi *et al*. 2005, Machtinger & Racowsky 2013).

When logistic regression was used to identify the importance of each factor, the type of endometrial preparation for FET cycles and the embryo quality were shown to be the most important factors for a risk of pregnancy loss. Overall the results of this study demonstrate that when consulting patients with RPL, the option of PGT-A might still result in a disappointing outcome since there are other factors that are often overlooked that might be useful in providing a risk for embryo loss.

This study has some limitations including the retrospective nature and the existence of other possible confounders that were not analysed. Follow-up validation and inclusion of factors that were not available in this study but can be associated with miscarriage rates, such as paternal age and sperm DNA fragmentation, will further highlight the importance of a holistic approach when consulting patients. In addition there is a lack on data on the number of miscarriages. With further validation, it would also be clear whether selecting against uneven blastomeres would be clinically useful in reducing the euploid loss risk. Prospective studies should also focus on testing endometrial preparation types in homogeneous groups regarding to establish if the increased miscarriage rates in HRT cycles are due to the cycle type or reproductive function. The study has several strengths, such as the large number of PGT-A tested embryos originating from a single centre, using the same laboratory conditions. Furthermore, the evaluation of embryo quality from a single senior embryologist, avoiding therefore inter-observer variability.

When a pregnancy loss occurs following a euploid embryo transfer after controlling for all anatomical and endocrine disorders, explaining its reasons to patients is often one of the biggest challenges for clinicians. To our knowledge, this is the largest study that examines a series of clinical, cycle, and embryological factors that can affect the outcome of a pregnancy following transfer of a single euploid embryo.

## Conclusion

Even though PGT-A is widely used to avoid pregnancy losses, it is still a possible outcome. The type of endometrial preparation, the presence of uneven blastomeres, embryo quality, and the number of previous FET cycles have been identifying as factors that can impact pregnancy outcomes following transfer of a single euploid embryo. This knowledge can be used when consulting patients. Upon validation, consideration of these factors can be used for correction of amenable factors prior to initiation of treatment and embryo selection with the ultimate goal of increasing the chance of achieving LB. Furthermore, this knowledge can be used when consulting the patients.

## Declaration of interest

The authors have no relevant financial or non-financial interests to disclose. GL is an Associate Editor of *Reproduction and Fertility*. GL was not involved in the review or editorial process for this paper, on which he is listed as an author.

## Funding

This work did not receive any specific grant from any funding agency in the public, commercial, or not-for-profit sector.

## Ethical approval

Ethical approval was waived by the local Ethics Committee of Istanbul Memorial Hospital in view of the retrospective nature of the study (no. 24.12.2021/008).

## Author contributions

All authors contributed to the study conception and design. Material preparation and data collection were performed by BY, YK, and HY. The first draft of the manuscript was written by BY, GO, INBD, and AA and the final draft of the manuscript was prepared by GL and MS. All authors read and approved the final version of the manuscript.

## Patient consent

Informed, written consent was obtained from all individual participants included in the study accepting controlled ovarian stimulation, oocyte pick-up procedure, freeze-all, PGT-A, and embryo transfer procedure.

## Data availability

The datasets generated and/or analysed during the current study are not publicly available due to patient privacy and hospital policy but are available from the corresponding author on reasonable request.
